# Comparison of statistical models to estimate parasite growth rate in the induced blood stage malaria model

**DOI:** 10.1186/s12936-017-1999-1

**Published:** 2017-08-25

**Authors:** Leesa F. Wockner, Isabell Hoffmann, Peter O’Rourke, James S. McCarthy, Louise Marquart

**Affiliations:** 1 QIMR Berghofer Medical Research Institute, Brisbane, QLD 4006 Australia; 2 Institute of Medical Biostatistics, Epidemiology and Informatics, Medical Center of Johannes Gutenberg University, 55101 Mainz, Germany; 3 School of Medicine, University of Queensland, Brisbane, QLD 4072 Australia; 4 Q-Pharm Pty Ltd, Brisbane, QLD 4006 Australia

**Keywords:** Statistical modelling, Fixed intercept regression, Parasite growth rate, Controlled human malaria infection, Induced blood stage malaria, Clinical trial

## Abstract

**Background:**

The efficacy of vaccines aimed at inhibiting the growth of malaria parasites in the blood can be assessed by comparing the growth rate of parasitaemia in the blood of subjects treated with a test vaccine compared to controls. In studies using induced blood stage malaria (IBSM), a type of controlled human malaria infection, parasite growth rate has been measured using models with the intercept on the y-axis fixed to the inoculum size. A set of statistical models was evaluated to determine an optimal methodology to estimate parasite growth rate in IBSM studies.

**Methods:**

Parasite growth rates were estimated using data from 40 subjects published in three IBSM studies. Data was fitted using 12 statistical models: log-linear, sine-wave with the period either fixed to 48 h or not fixed; these models were fitted with the intercept either fixed to the inoculum size or not fixed. All models were fitted by individual, and overall by study using a mixed effects model with a random effect for the individual.

**Results:**

Log-linear models and sine-wave models, with the period fixed or not fixed, resulted in similar parasite growth rate estimates (within 0.05 log_10_ parasites per mL/day). Average parasite growth rate estimates for models fitted by individual with the intercept fixed to the inoculum size were substantially lower by an average of 0.17 log_10_ parasites per mL/day (range 0.06–0.24) compared with non-fixed intercept models. Variability of parasite growth rate estimates across the three studies analysed was substantially higher (3.5 times) for fixed-intercept models compared with non-fixed intercept models. The same tendency was observed in models fitted overall by study. Modelling data by individual or overall by study had minimal effect on parasite growth estimates.

**Conclusions:**

The analyses presented in this report confirm that fixing the intercept to the inoculum size influences parasite growth estimates. The most appropriate statistical model to estimate the growth rate of blood-stage parasites in IBSM studies appears to be a log-linear model fitted by individual and with the intercept estimated in the log-linear regression. Future studies should use this model to estimate parasite growth rates.

**Electronic supplementary material:**

The online version of this article (doi:10.1186/s12936-017-1999-1) contains supplementary material, which is available to authorized users.

## Background

The Global Technical Strategy for Malaria 2016–2030 aims to reduce the incidence of new malaria cases by at least 90% by 2030 [1]. Among the tools that could assist achieving this goal are vaccines that prevent malaria parasite growth in the blood, that is, when the parasite is in the blood stage of its lifecycle. A reliable method to assess the efficacy of blood-stage vaccines is fundamental to decide which candidates are worth further development. A standard methodology to evaluate the activity of blood-stage vaccines is measuring the parasite growth rate, from which the parasite multiplication rate (PMR) can be derived [2]. Parasite growth rate can be estimated in controlled human malaria infection studies [3, 4].

The induced blood stage malaria (IBSM) model is a type of controlled human malaria infection in which subjects are inoculated with blood-stage parasites. The inoculum size can be controlled and therefore all subjects in the study can be inoculated safely and uniformly [5]. Parasitaemia in the blood of subjects is monitored by quantitative PCR (qPCR) [6], which allows timely data collection from study subjects to estimate parasite growth rate at low levels of quantitation. Efficacy of blood-stage vaccines can be assessed in IBSM studies by determining the reduction in parasite growth rate in the treatment group compared to the control group. As a result, the IBSM model is been increasingly used to test the efficacy of blood-stage vaccine candidates [7, 8].

Statistical approaches to estimate parasite growth rate include either log-linear or sine-wave models fitted to the log_10_ parasite counts over time [4, 9]. The models used in published IBSM studies [7, 8, 10] have fixed the intercept on the y-axis to the inoculum size administered to subjects in a given cohort as determined by qPCR. Given that the first parasite counts are only detected by qPCR around 4 days after inoculation, the intercept is fixed to the inoculum size by extrapolating the parasitaemia curve to day 0, which is outside of the range of available data, that is, from day 4 to day 7 or 8, the day when the first anti-malarial treatment is typically given. This extrapolation presumes that parasites would grow log-linearly from day 0 to day 4 and at the same rate as in the measured growth period. Extrapolating the available data from day 4 to day 0 generates a highly influential point (a point of high leverage) assumed to be measured without error, which could bias estimation of the parasite growth rate.

Additionally, models with the intercept fixed to the inoculum size assume that the starting circulating parasitaemia equals the inoculum size and is known for each individual. Although the preparation of the inoculum can be standardised and its size quantified, the actual number of viable parasites introduced into the blood stream of each subject cannot be known with certainty and may be influenced by a number of factors. For example, the time interval between thawing of parasite vials and injection into subjects varies, both within a cohort and between cohorts. Thus, the loss of parasite viability over time would result in some variation in the inoculum size administered to each subject. Moreover, variations in the process of inoculum preparation may result in differences in the inoculum size between cohorts. Hence inoculum size is a controlled variable rather than a constant.

An accurate estimation of the parasite growth rate is paramount to assess the efficacy of vaccine candidates against malaria. In this report, data from three published IBSM studies in which the parasite growth rates were estimated using models with the intercept fixed to the inoculum size was re-analysed [7, 8, 10]. A set of statistical models was fitted to the published data, including both a fixed and a non-fixed intercept approach, and the estimated parasite growth rates compared.

## Methods

### Studies and subjects analysed

Data from three previously published studies in which subjects were inoculated with *Plasmodium falciparum* 3D7 IBSM was analysed [7, 8, 10]. A total of 40 subjects were analysed: five subjects from the Sanderson et al. study; eight subjects from the Duncan et al. study (five vaccinated with AMA1-C1/Alhydrogel+ CPG 7909 and three unvaccinated controls); and 27 subjects from the Payne et al. study (12 vaccinated with FMP2.1/AS01 and 15 unvaccinated controls). Details about study design, and inclusion and exclusion criteria can be found in the original publications [7, 8, 10].

### Description of statistical models

Data were modelled using 12 statistical models to estimate the parasite growth rate. The models are described below and summarized in Table 1.

**Table 1:** Statistical models used in this study

Model no^a^	Type of model	Period^b^	Intercept^c^
1 and 7	Log-linear	NA	Fixed intercept
2 and 8	Log-linear	NA	Non-fixed intercept
3 and 9	Sine-wave	Fixed period	Fixed intercept
4 and 10	Sine-wave	Fixed period	Non-fixed intercept
5 and 11	Sine-wave	Non-fixed period	Fixed intercept
6 and 12	Sine-wave	Non-fixed period	Non-fixed intercept

*NA* not applicable

^a^Models 1–6 were fitted by individual. Models 7–12 were fitted overall by study with a mixed effects model with a random effect for the individual

^b^Fixed-period models had the period fixed to 48 h

^c^Fixed-intercept models had the intercept fixed to the inoculum size provided in the original publications

The parasite growth rate can be expressed as the PMR standardized to 48 h as given by:

$${\text{PMR}}_{48} = 10^{(2m)} ,$$

where *m* is the parasite growth rate, and 2 days is the 48-h period. For purposes of reporting, the primary parameter (*m)* rather than the derived PMR is presented. Log-linear and sine-wave models were used to estimate the parasite growth rate, *m*.

#### Log-linear model

The log-linear model used to estimate the parasite growth rate can be described as follows:

$${ \log }_{ 10} \left( Y \right) \, = a + m \times time,$$

where *Y* = parasites per mL measured by qPCR at multiple times from inoculation to first anti-malarial treatment, *a* = intercept, *m* = parasite growth rate, and *time* = days from inoculation. This model was fitted by individual using simple linear regression, or overall by study using a linear mixed effects model with a random effect for *a*, assumed to be independent for each individual. Finally, the model was fitted either fixing or not fixing the intercept to the inoculum size. For the mixed effects models, the intercept was fixed to the inoculum size for the mean of the random effects.

#### Sine-wave model

The sine-wave model used to estimate the parasite growth rate can be described as follows [4, 9]:

$$\log_{10} (Y) = a + m \times time + c \times \sin \;((2 \times \pi /period) \times time + k),$$

where *Y* = parasites per mL measured by qPCR, *a* = intercept, *m* = parasite growth rate, *c* = amplitude of the sine wave, *period* = length of a parasite life-cycle in days, *time* = days from inoculation, and *k* = phase shift in sine wave. The model was fitted by individual using non-linear regression, or overall by study using a non-linear mixed effects model with a random effect for *a*, assumed to be independent for each individual. The models were fitted either fixing the period to the commonly used period length of 48 h or allowing the fitting procedure to estimate the period. Finally, the by individual models were fitted either fixing the intercept to the inoculum size or estimating the intercept. Similarly, the mixed effects models were fitted by fixing the mean of the random effects to the inoculum size or estimating it as part of the model.

### Inoculum size

The intercept of fixed-intercept models was fixed to the reported inoculum size in each study: ~1800 viable parasites in Sanderson et al. [10], ~250 viable parasites in Duncan et al. [7] and ~690 viable parasites in Payne et al. [8]. The size of the inocula was converted to parasites per millilitre by assuming the volume of blood for an individual was 5000 mL. In the instance of Payne et al. [8], subjects’ body weights were known, and hence more accurate blood volumes could be derived (Additional file 1). The body weight of subjects from the Sanderson et al. and Duncan et al. studies was not available.

### Statistical analyses

Data were processed as detailed in the relevant publications. The parasite growth rate estimated in this study were compared to the estimates reported in the original publications using the same model: Payne et al. [8] fitted log-linear models, whereas Duncan et al. [7] and Sanderson et al. [10] fitted sine-wave models with the period fixed to 48 h. In all three studies, the authors fitted the models by individual and fixed the intercept to the inoculum size. The average parasite growth rate and confidence of interval (CI) was calculated for Duncan et al. and Payne et al. studies by averaging the pooled results from the vaccine and control subjects in each of the studies (n = 8 for Duncan et al., n = 27 for Payne et al.).

The average parasite growth rates estimated for each study are presented along with their standard deviation (SD) and 95% CI for individual models, and the parasite growth rates for each study along with the standard error (SE) and 95% CI for the mixed effects models. The SD across studies was calculated from the average parasite growth rate values estimated for each of the three studies.

Coefficients of multiple determinations (R^2^) for the individual log-linear models with non-fixed intercept were calculated as 1-RSS/TSS, where RSS and TSS are the residual and total sum of squares, respectively. In linear regression, the TSS is corrected for the mean in non-fixed intercept models, but not in fixed-intercept models. To give comparable results, TSS was corrected for the mean in fixed-intercept models as described by Gordon [11].

All models were fitted using the program package R [12], version 3.2.2, and the package *nlme*, version 3.1 [13].

## Results

### Models fitted by individual

Average parasite growth rates estimated fitting models by individual (models 1–6) to data from three previous IBSM studies [7, 8, 10] are presented in Table 2. For the log-linear models, individual values are given in Additional file 1, and the PMR estimates in Additional file 2. The parasite growth rate estimated by Payne et al. [8] was replicated using a log-linear model with the intercept fixed (average parasite growth rate 0.50, 95% CI 0.48–0.52). However, exact replication of the parasite growth rates reported by Sanderson et al. [10] and Duncan et al. [7] using a sine-wave model with the intercept and the period fixed was not achieved. Sanderson et al. reported an average parasite growth rate of 0.66 (95% CI 0.53–0.79), whereas this study estimated a parasite growth rate of 0.63 (95% CI 0.58–0.69). Duncan et al. estimated a parasite growth rate of 0.61 (95% CI 0.56–0.66), whereas this study estimated a parasite growth rate of 0.52 (95% CI 0.49–0.54). However, individual body weights for these latter two studies were not available.

**Table 2:** Average parasite growth rate estimates for models fitted by individual (models 1–6)

Model used	Parameter estimated	Sanderson et al.		Duncan et al.		Payne et al.		SD across studies fixed/non-fixed intercept model
		Fixed intercept (n = 5)	Non-fixed intercept (n = 5)	Fixed intercept (n = 8)^a^	Non-fixed intercept (n = 8)^b^	Fixed intercept (n = 27)^c^	Non-fixed intercept (n = 27)^c^	
Log-linear	*m* (SD) [95% CI]	0.64 (0.064) [0.58–0.69]	0.72 (0.131) [0.61–0.84]	0.52 (0.039) [0.49–0.55]	0.71 (0.128) [0.62–0.80]	*0.50* (*0.049*) [*0.48–0.52*]	0.68 (0.097) [0.65–0.72]	0.073/0.021
Sine-wave (fixed period)	*m* (SD) [95% CI]	*0.63* (*0.065*) [*0.58–0.69*]	0.69 (0.197) [0.52–0.86]	*0.52* (*0.041*) [*0.49–0.54*]	0.73 (0.104) [0.65–0.81]	0.50 (0.047) [0.48–0.52]	0.70 (0.135) [0.65–0.75]	0.073/0.021
Sine-wave (non-fixed period)	*m* (SD) [95% CI]	0.62 (0.059) [0.57–0.67]	0.74 (0.122) [0.64–0.85]	0.52 (0.045) [0.49–0.55]	0.76 (0.055) [0.71–0.80]	0.50 (0.044) [0.48–0.51]	0.72 (0.112) [0.68–0.76]	0.067/0.019

Fixed intercept models had the intercept fixed to the inoculum size, whereas non-fixed intercept models estimated the intercept as part of model fitting. Sine-wave models were fitted either with the period fixed to 48 h (fixed period), or with the period not fixed (non-fixed period). The parasite growth rate estimate (*m*) is given as average across individuals in a study. The values highlighted in italic correspond with the models used to fit the data in the original reports

*m* parasite growth rate estimate, *SD* standard deviation, *CI* confidence interval

^a^Except for sine-wave (non-fixed period) model, n = 7

^b^Except for sine-wave (fixed period) model, n = 7; sine-wave (non-fixed period) model, n = 6

^c^Except for sine-wave (non-fixed period) model, n = 26

Log-linear models, as well as sine-wave models fitted with the period fixed to 48 h or non-fixed, resulted in almost identical estimates of average parasite growth rates (within 0.05 log_10_ parasites per mL/day) and SD (within 0.07) in the three studies analysed, regardless of fixing or not fixing the intercept (Table 2).

In contrast, fixing the intercept had a substantial effect on average parasite growth rate estimates in all fitted models. Fixed-intercept models estimated lower average parasite growth rate values than the corresponding non-fixed intercept models (Table 2). The average parasite growth rate estimate was 0.17 log_10_ parasites per mL/day (range 0.06–0.24) lower for fixed-intercept models. Moreover, variability (SD) of the parasite growth rate estimates between the three studies analysed was substantially higher (3.5-fold) with fixed-intercept models than with non-fixed intercept models. This increase in variability was observed in log-linear models (SD: 0.073 vs 0.021), sine-wave models with fixed period (SD: 0.073 vs 0.021) and sine-wave models with non-fixed period (SD: 0.067 vs 0.019). However, the variability of the parasite growth rate estimates within a study appears to be lower in fixed-intercept models (Table 2). Non-fixed intercept models had a higher R^2^ in all log-linear fits than the corrected R^2^ value for the fixed-intercept models (Additional file 1).

### Mixed effects models fitted overall by study

Parasite growth rates estimated fitting models overall by study (models 7–12) to data from three previous IBSM studies [7, 8, 10] are presented in Table 3. Patterns in estimates of parasite growth rates across the different statistical models and studies were similar for the mixed effects models fitted overall by study and models fitted by individual (Tables 2, 3). An exception was the parasite growth rate estimated for the study from Payne et al. with a sine-wave model with the period not fixed and the intercept fixed, which was 0.50 (95% CI 0.48–0.51) if the parasite growth rate was estimated by individual, and 0.67 (95% CI 0.65–0.70) when estimated overall by study.

**Table 3:** Parasite growth rate estimates for models fitted overall by study using mixed effects models (models 7–12)

Model used	Parameter estimated	Sanderson et al.		Duncan et al.		Payne et al.		SD across studies fixed/non-fixed intercept model
		Fixed intercept (n = 5)	Non-fixed intercept (n = 5)	Fixed intercept (n = 8)	Non-fixed intercept (n = 8)	Fixed intercept (n = 27)	Non-fixed intercept (n = 27)	
Log-linear	*m* (SE) [95% CI]	0.63 (0.025) [0.58–0.68]	0.67 (0.084) [0.50–0.84]	0.53 (0.013) [0.50–0.55]	0.69 (0.055) [0.58–0.80]	0.52 (0.010) [0.50–0.54]	0.66 (0.024) [0.61–0.71]	0.061/0.017
Sine-wave (fixed period)	*m* (SE) [95% CI]	0.63 (0.024) [0.58–0.67]	0.67 (0.083) [0.51–0.83]	0.53 (0.015) [0.50–0.56]	0.74 (0.036) [0.67–0.81]	0.52 (0.010) [0.50–0.54]	0.68 (0.022) [0.63–0.72]	0.059/0.040
Sine-wave (non-fixed period)	*m* (SE) [95% CI]	0.62 (0.024) [0.58–0.67]	0.71 (0.068) [0.58–0.84]	0.53 (0.016) [0.50–0.56]	0.73 (0.035) [0.66–0.80]	0.67 (0.014) [0.65–0.70]	0.70 (0.015) [0.67–0.72]	0.071/0.018

Fixed-intercept models had the intercept fixed to the inoculum size, whereas non-fixed-intercept models estimated the intercept as part of model fitting. Sine-wave models were fitted either with the period fixed to 48 h (fixed period), or with the period not fixed (non-fixed period). The parasite growth rate estimate (*m*) overall by study was estimated by the mixed effects model

*m* parasite growth rate estimate, *SD* standard deviation, *SE* standard error, *CI* confidence interval

## Discussion

In the present study, 12 different statistical models were fitted to data from three previously published studies to identify the optimal model for estimation of the parasite growth rate in IBSM studies. The analyses show that fitting log-linear and sine-wave models to data without fixing the intercept to the inoculum size results in smaller variability of the parasite growth rate estimates between studies than fitting models with the intercept fixed. This decrease in variability was observed in models fitted by individual and overall by study. The results of this study suggest that the parasite growth rate is similar regardless of inoculum size, which is consistent with the understanding of the biology of parasite growth.

The variability of the parasite growth rate estimates for models fitted by individual within a study is lower in fixed than non-fixed intercept models, which may be an artefact due to the high leverage of the fixed intercept. When the intercept is fixed to the inoculum size, the parasite growth rate is forced to be similar for all subjects within a study, hence reducing the variability across individual parasite growth rate estimates. Variability of the parasite growth rate is crucial for calculation of sample size of IBSM studies: the lower the variability, the smaller the required sample size. Therefore, it is important that variability of the parasite growth rate is correctly estimated and generalizable to the larger population, that is, not study specific.

The error associated with the parasite growth rate estimates for models fitted overall by study using mixed effects models appears also lower for fixed than for non-fixed intercept models. However, as detailed in Marquardt et al. [14], comparing the error from fixed and non-fixed intercept models estimated using mixed effects is not appropriate.

The results presented in this report confirm previously reported findings that parasite growth rate estimates are similar in log-linear and sine-wave models [15] and that log-linear models are functionally equivalent to sine-wave models when evaluating parasite growth rate. Sine-wave models provide useful additional information on the periodicity and amplitude of the in vivo growth of *P. falciparum*. For computational purposes, fixing the intercept to the inoculum size can facilitate modelling as estimating one fewer parameter can reduce difficulties with model convergence. This is particularly true for sine-wave modelling, where even after fixing the period to 48 h the model will still require five or more data points per subject to estimate all parameters. Nevertheless, sine-wave, non-linear mixed effects models allow all data points to be included, even if fewer than five data points are available for some subjects.

Modelling the data by individual or overall by study had minimal effect on parasite growth rate estimates in malaria-naïve subjects. Mixed effect models used to fit data overall by study combine data with appropriate weights across individuals to estimate an overall parasite growth rate. In models fitted by individual, averages of the individual fits are an unweighted version of the same analysis. Hence, it is not surprising that the analyses performed either by individual or overall by study give very similar estimates of the in vivo growth rate of the *P. falciparum* 3D7 parasite. Based on simplicity and greater flexibility, the individual fits are preferred over overall study fits. Moreover, individual fits allow investigation of individual immune factors, which are of interest in vaccine trials. However, if the subjects differ greatly in number of data points or have very few points available for modelling because of logistical issues, a weighted average of the individual fits should be considered.

A number of biological reasons further support the rationale for not fixing the intercept when estimating the parasite growth rate. Fixed intercept models assume that the number of viable parasites in the inoculum is constant, both between study subjects in an individual cohort and across studies. However, there is a paucity of experimental data to support this hypothesis. Moreover, a range of sources indicate that parasites may grow at different rates in different subjects, depending on factors such as the subject age, immunological response and red cell factors that may influence parasite replication [16–18]. Thus, extrapolating data from day 4, when parasites are initially detected by qPCR, to day 0, may introduce a confounding effect that is numerically substantial and lacking in biologic plausibility. By not fixing the intercept, this potential confounding effect is accounted for. Therefore, parasitaemia at day 0 for individual study subjects is more accurately estimated using non-fixed intercept models.

The analyses presented in this report were slightly different compared with the original reports. Whether the intercept was fixed to the same inoculum size as in the original reports is not certain. However, since this study closely reproduces the parasite growth rate estimates reported in each of the original publications, the differences between the analyses are not critical for the conclusions of this study.

## Conclusions

The most appropriate model to estimate the parasite growth rate in IBSM studies appears to be a log-linear model fitted by individual and with the intercept not fixed to the inoculum size. Parasite growth rates estimated with this model are less variable across studies than the equivalent model with the intercept fixed to the inoculum size. Importantly, using non-fixed intercept models to estimate the parasite growth rate would allow comparison of parasite growth rate estimates between studies, resulting in a more powerful assessment of efficacy of anti-malarial vaccines. Extending the comparison of vaccine efficacy across different research centres would accelerate the development of efficient vaccines against malaria by improving the design of clinical trials that test interventions aimed to eradicate malaria. Moreover, the parasite growth rate could also be used to evaluate the efficacy of chemoprophylaxis that target the blood-stage parasite.

## Additional files

**Additional file 1.** Parasite growth rate and coefficient of multiple determinations of log-linear models.

**Additional file 2.** Average parasite multiplication rate estimates for log-linear models fitted by individual.
