# Supplementary material for: Comparison of statistical models to estimate parasite growth rate in the induced blood stage malaria model
Source: Malar J. 2017 Aug 25;16:352. doi: 10.1186/s12936-017-1999-1 (PMC5574106; doi:10.1186/s12936-017-1999-1)
Supplement: Supplementary file 1 — Additional file 1. Parasite growth rate and coefficient of multiple determinations of log-linear models. [file 12936_2017_1999_MOESM1_ESM.docx]

**Additional file 1.** **Parasite growth rate and coefficient of multiple determinations of log‑linear models**

|  |  | **Fixed intercept model** | | **Non-fixed intercept model** | |
| --- | --- | --- | --- | --- | --- |
| **Subject No.^a^** | **Intercept value^b^**  **log_10_ (viable parasites/blood volume [mL])** | ***m*** | **Corrected R^2^** | ***m*** | **R^2^** |
| **Sanderson et al.** | | | | | |
| B1 | log_10_(1800/5000) | 0.586 | 0.653 | 0.736 | 0.683 |
| B2 | log_10_(1800/5000) | 0.566 | 0.440 | 0.527 | 0.443 |
| B3 | log_10_(1800/5000) | 0.632 | 0.637 | 0.740 | 0.651 |
| B4 | log_10_(1800/5000) | 0.669 | 0.719 | 0.896 | 0.771 |
| B5 | log_10_(1800/5000) | 0.726 | 0.814 | 0.717 | 0.814 |
| **Duncan et al.** |  |  |  |  |  |
| C1 | log_10_(250/5000) | 0.480 | 0.483 | 0.830 | 0.588 |
| C2 | log_10_(250/5000) | 0.500 | 0.794 | 0.755 | 0.898 |
| C3 | log_10_(250/5000) | 0.568 | 0.852 | 0.786 | 0.926 |
| V1 | log_10_(250/5000) | 0.542 | 0.726 | 0.783 | 0.805 |
| V2 | log_10_(250/5000) | 0.542 | 0.638 | 0.730 | 0.684 |
| V3 | log_10_(250/5000) | 0.519 | 0.787 | 0.772 | 0.884 |
| V4 | log_10_(250/5000) | 0.455 | 0.412 | 0.446 | 0.412 |
| V5 | log_10_(250/5000) | 0.552 | 0.699 | 0.595 | 0.703 |
| **Payne et al.** |  |  |  |  |  |
| AMA1-1 | log_10_(690/5222) | 0.484 | 0.657 | 0.668 | 0.712 |
| AMA1-2 | log_10_(690/5250) | 0.554 | 0.617 | 0.809 | 0.687 |
| AMA1-3 | log_10_(690/6230) | 0.541 | 0.803 | 0.598 | 0.811 |
| AMA1-4 | log_10_(690/2940) | 0.557 | 0.791 | 0.729 | 0.839 |
| AMA1-5 | log_10_(690/5950) | 0.503 | 0.641 | 0.568 | 0.650 |
| AMA1-6 | log_10_(690/6300) | 0.437 | 0.664 | 0.577 | 0.706 |
| AMA1-7 | log_10_(690/6251) | 0.451 | 0.832 | 0.587 | 0.881 |
| AMA1-8 | log_10_(690/5460) | 0.511 | 0.775 | 0.651 | 0.814 |
| AMA-9 | log_10_(690/4417) | 0.425 | 0.556 | 0.461 | 0.560 |
| AMA-10 | log_10_(690/8540) | 0.517 | 0.741 | 0.848 | 0.877 |
| AMA1-11 | log_10_(690/5894) | 0.492 | 0.680 | 0.787 | 0.796 |
| AMA1-12 | log_10_(690/4970) | 0.558 | 0.704 | 0.802 | 0.778 |
| C1 | log_10_(690/5313) | 0.554 | 0.598 | 0.684 | 0.620 |
| C2 | log_10_(690/6132) | 0.475 | 0.810 | 0.694 | 0.905 |
| C3 | log_10_(690/4620) | 0.538 | 0.768 | 0.672 | 0.801 |
| C4 | log_10_(690/4515) | 0.412 | 0.776 | 0.646 | 0.896 |
| C5 | log_10_(690/4690) | 0.526 | 0.670 | 0.784 | 0.754 |
| C6 | log_10_(690/5355) | 0.493 | 0.725 | 0.851 | 0.885 |
| C7 | log_10_(690/6930) | 0.554 | 0.689 | 0.717 | 0.728 |
| C8 | log_10_(690/5026) | 0.417 | 0.723 | 0.569 | 0.779 |
| C9 | log_10_(690/4550) | 0.486 | 0.769 | 0.620 | 0.808 |
| C10 | log_10_(690/3696) | 0.497 | 0.874 | 0.606 | 0.904 |
| C11 | log_10_(690/5110) | 0.524 | 0.757 | 0.763 | 0.842 |
| C12 | log_10_(690/5173) | 0.586 | 0.711 | 0.717 | 0.737 |
| C13 | log_10_(690/4046) | 0.527 | 0.810 | 0.658 | 0.845 |
| C14 | log_10_(690/4200) | 0.498 | 0.713 | 0.755 | 0.812 |
| C15 | log_10_(690/5418) | 0.426 | 0.718 | 0.606 | 0.790 |

*m*: parasite growth rate estimate; R^2^: coefficient of multiple determinations.

^a^ All subjects were inoculated with IBSM. Subjects in Duncan et al. were either previously vaccinated with AMA1/C1 Alhydrogel+CPG 7909 (n=5, V1 to V5) or unvaccinated controls (n=3, C1 to C3). Subjects in Payne et al. were either previously vaccinated with FMP2.1/AS01 (n=12, AMA1-1 to AMA1-12) or unvaccinated controls (n=15, C1 to C15).

^b^ The intercept was fixed to the number of viable parasites present in the inoculum divided by the blood volume of the subject (mL). The estimated number of viable parasites was 1800 for Sanderson et al., 250 for Duncan et al., and 690 for Payne et al. Subjects’ blood volume was assumed to be 5000 mL for Sanderson et al. and Duncan et al. Body weights were provided for Payne et al. and hence more accurate estimates of blood volumes were calculated.
