# Supplementary material for: Comparison of statistical models to estimate parasite growth rate in the induced blood stage malaria model
Source: Malar J. 2017 Aug 25;16:352. doi: 10.1186/s12936-017-1999-1 (PMC5574106; doi:10.1186/s12936-017-1999-1)
Supplement: Supplementary file 2 — Additional file 2. Average parasite multiplication rate estimates for log-linear models fitted by individual. [file 12936_2017_1999_MOESM2_ESM.docx]

Additional file 2. Average parasite multiplication rate estimates for log‑linear models fitted by individual^a^.

| **Study** | **Fixed intercept model** | **Non- fixed intercept model** |
| --- | --- | --- |
| **Sanderson et al. (n=5)** | 19.05 [14.45-23.99] | 27.54 [16.60-47.86] |
| **Duncan et al. (n=8)** | 10.96 [9.55-12.59] | 26.30 [17.38-39.81] |
| **Payne et al. (n=27)** | 10.00 [9.12-10.96] | 22.91 [19.95-27.54] |

^a^ The fixed intercept model had the intercept fixed to the inoculum size, whereas non-fixed intercept model estimated the intercept as part of model fitting. The parasite multiplication rate estimate is given as average across individuals in a study. The 95% confidence interval is provided in brackets.
